# Researches on the Medicinal Properties and Applications of Nitrous Oxide, Protoxide of Nitrogen, or Laughing Gas

**Published:** 1865-12

**Authors:** 


### Book Notices.

Researches on the Medicinal Properties and Applications of Nitrous Oxide, Protoxide of Nitrogen, or Laughing Gas. By Geo. J. Ziegler, M.D., Physician to Philadelphia Hospital, &c., &c. Philadelphia: J. B. Lippincott & Co. 1865.

This is a very neatly printed little volume of 66 pages, in which the author discusses the physiological, pathological, and therapeutic properties and applications of nitrous oxide. The book is full of suggestions of an interesting character, and will abundantly repay for the time spent in perusing it, but is very deficient in facts or cases calculated to sustain and illustrate the practical application and correctness of the suggestions made. We have long entertained the opinion that the nitrous oxide gas afforded a most valuable remedy in sustaining the susceptibility of the tissues and the integrity of the blood, in the lower grades of typhoid and typhus fevers, as well as in the pernicious form of intermittents, and in cholera. But owing to the inconvenience of preparation and administration, we have never adequately tested our opinion by practical application. We hope this little work of Dr. Ziegler will so far excite the attention of the profession, as to cause the medicinal properties and value of the nitrous oxide gas to be fully tested, especially in cholera, if that disease should prevail in our country the coming spring and summer.

The work is for sale by W. B. Keen & Co., Lake Street, Chicago.

